# PHScaffolding: a hypergraph clustering and dual-weight integration strategy for scaffolding with Pore-C reads

**DOI:** 10.1093/bib/bbag003

**Published:** 2026-01-22

**Authors:** Quan Su, Junwei Luo, Fei Guo

**Affiliations:** School of Software, Henan Polytechnic University, No. 2001, Shiji Road, High-tech Zone, Jiaozuo, Henan, 454003, China; School of Software, Henan Polytechnic University, No. 2001, Shiji Road, High-tech Zone, Jiaozuo, Henan, 454003, China; School of Computer Science and Engineering, Central South University, No. 932, Lushan South Road, Yuelu District, Changsha, Hunan, 410083, China

**Keywords:** scaffolding, Pore-C, hypergraph, Louvain

## Abstract

Genome assembly aims to construct chromosome-level genome sequences, with scaffolding serving as a critical step, the accuracy of which highly depends on the quality of the input data. Although both Hi-C and Pore-C technologies are used to study genomic 3D structures, Pore-C demonstrates irreplaceable advantages in high-precision assembly due to its ability to capture long-range information and provide multi-fragment interaction information. However, most current scaffolding methods primarily rely on Hi-C data, which is limited by the inherent constraints of the technology, resulting in deficiencies in assembly continuity and accuracy. We propose a scaffolding method based on Pore-C data, named PHScaffolding. This method constructs a hypergraph by leveraging alignment information from Pore-C reads to capture multi-way interactions among contigs. A dedicated weighting scheme for hyperedges is also introduced. Subsequently, PHScaffolding applies the Louvain algorithm to cluster the hypergraph, aiming to group contigs originating from the same chromosome. Finally, for contigs within each cluster, the method employs a novel strategy to orient and order them based on Pore-C read alignments, thereby generating chromosome-level scaffolds. Evaluations on HG002, GM12878, and *Arabidopsis thaliana* contig datasets demonstrate that PHScaffolding achieves strong performance and robustness in terms of NA50, NGA50, and misassembly rates. Comparative experiments show that it outperforms traditional Hi-C-based scaffolding methods. The source code of PHScaffolding is available at https://github.com/Suquana/PHScaffolding.

## Introduction

Genome sequences form the foundation for studies on species growth, development, morphology, disease, and lifespan. With advancements in technology, genome assembly has become a key focus in the field of genomics research [1–5]. Genome assembly refers to the process of reconstructing complete and accurate genome sequences from raw sequencing data, and high-quality chromosome-level genome sequences are an important foundation for downstream genomics research [6–8]. In this context, scaffolding, one of the key steps in genome assembly, plays a crucial role.

In the field of genome assembly, traditional scaffolding methods are primarily based on second-generation sequencing technologies that generate short reads or long reads. Short-read-based methods include SHARCGS [9], Velvet [10], and ALLPATHS2 [11], while long-read-based methods encompass SSPACE-LongRead [12], LINKS [13], npScarf [14], and SLR [15]. However, short reads are relatively fragmented and struggle to span repetitive regions [16], while long reads suffer from higher sequencing costs and elevated error rates [17]. Consequently, neither approach can efficiently assemble chromosome-level genome sequences.

Hi-C technology [18] derived from chromosome conformation capture (3C), was initially used to investigate the 3D spatial structure of genomes. By leveraging long-range interaction information between genomic fragments, it has been widely adopted for assembling chromosome-level genome sequences [19].

Now, there exists many scaffolding methods based on Hi-C reads. 3D-deoxyribonucleic acid (DNA) [20] utilizes Hi-C long-range contact patterns to detect assembly errors in contigs and correct them, thereby enhancing accuracy. Subsequently, it iteratively anchors, orders, and orients the corrected contigs based on Hi-C contact frequency among contigs. Multiple iterations ultimately yield chromosome-level scaffolds. SALSA2 [21] detects assembly errors in contigs by identifying abrupt changes in Hi-C physical coverage patterns. After error correction, it constructs a hybrid graph combining Hi-C contact frequency and the assembly graph. Using a ‘best buddy’ weighting scheme, this approach then iteratively anchors, orders, and orients corrected contigs to assemble highly accurate chromosome-level scaffolds. YaHS [22] applies iterative greedy anchoring, ordering, and orienting—guided by inter-contig Hi-C contact frequency—to assemble chromosome-level scaffolds. EndHiC [23] enhances the signal-to-noise ratio by utilizing Hi-C links from contig ends and employs error correction, breakpoint detection, reciprocal best-hit filtering, and iterative optimization to achieve chromosome-scale scaffolding with high accuracy.

Although these methods can effectively assemble chromosome-level genome sequences, their accuracy remains inadequate when handling shorter contigs and repetitive regions [24]. In recent years, an emerging advanced sequencing technology, Pore-C [25], has been developed. Multiple studies have demonstrated Pore-C’s superior performance. Compared to Hi-C, Pore-C offers distinct advantages: (i) this technology integrates 3C with nanopore sequencing, providing interactions between multiple genomic fragments that better reflect actual genomic contexts. (ii) Pore-C read have longer length than Hi-C read. This longer read length facilitates traversing complex regions and repetitive regions within contigs, thereby enabling more efficient scaffolding [26–30].

To address this, we propose PHScaffolding, a Pore-C-based scaffolding method. This approach constructs a hypergraph to store contig relationships by parsing Pore-C alignment data. A hypergraph [31]—a specialized graph structure—allows hyperedges to connect any number of vertices (unlike conventional graphs where edges link only two vertices). This breaks the ‘edge = pairwise relationship’ constraint, enabling hyperedges to directly represent multi-way relationships. Additionally, we designed a geometrically balanced hyperedge weight calculation scheme tailored for this scenario. After hypergraph construction, the Louvain algorithm [32] is utilize to cluster the hypergraph, followed by variable-resolution binning to identify contig end regions. Two weighting schemes—terminal coverage and cosine similarity [33]—are then integrated to calculate the weights among contigs. Finally, the contigs are ordered, oriented, and connected into chromosome-scale scaffolds based on these weights.

We evaluated PHScaffolding on HG002 and GM12878 contig datasets using the QUAST [34] assessment tool. Compared to traditional Hi-C methods, PHScaffolding achieved superior results, demonstrating robust performance in NA50, NGA50, and misassembly metrics.

The primary contributions of this paper are:

(1) This method employs hypergraph to store the multi-way interaction contained in Pore-C read, and develops a new method to weight the hyper-edge. And the method adopts Louvain algorithm to find contigs from the same chromosome. Existing approaches commonly transfer Pore-C alignments to paired alignment, then adopt traditional scaffolding methods based on Hi-C reads.

(2) During the ordering and orientation phase, terminal regions of contigs are partitioned by size. Coverage weight and cosine similarity weight are integrated to synergistically ordering and orientation, improving both the accuracy and continuity of assembly results.

## Methods

**Figure 1:**
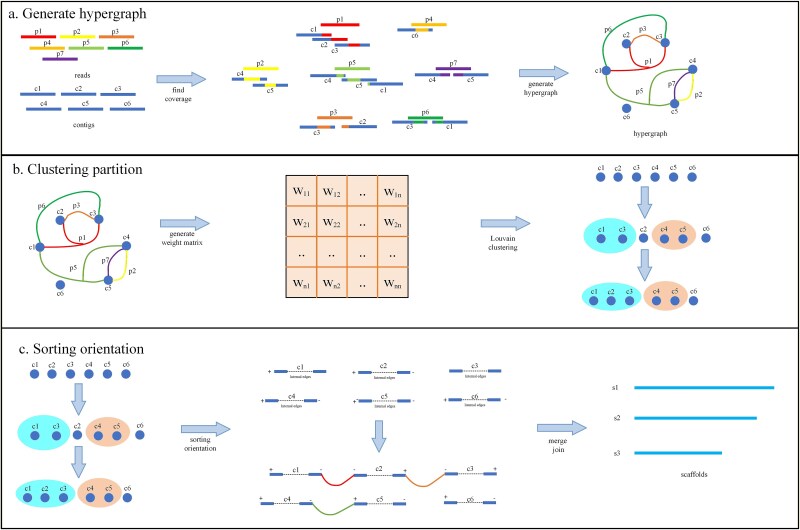
PHScaffolding pipeline. (a) Pore-C reads (p1–p7) are aligned to contigs (c1–c6), followed by the construction of a hypergraph based on the alignment information. (b) After hypergraph construction, edge weights are calculated and the Louvain algorithm is applied to partition the contigs. (c) Following partitioning, contigs are linearized into a single chain, then ordered, oriented, and finally assembled into complete scaffolds.

PHScaffolding is a Pore-C-based scaffolding method integrating hypergraph clustering and dual-weight ordering and orienting. The pipeline accepts Pore-C data and contigs as input, generating scaffold assemblies through four core steps. This workflow is illustrated in Fig. 1.

(1) Hypergraph construction: alignment data between Pore-C reads and contigs are analyzed to construct a hypergraph, where hyperedge weight are computed by a new strategy;
(2) Hypergraph clustering: contigs are clustered using the Louvain algorithm, where each cluster aims to contain the same contigs from the same chromosome;
(3) Ordering and orienting: within each cluster, contigs are ordered and oriented. By employing multi-scale contig terminal regions and a dual-weight integration strategy (coverage weight and cosine similarity weight), the interaction weight between nodes is calculated in the partitioned region. Subsequently, an initial ordering and orientation of contigs is performed based on the cosine similarity weight, following the highest-weight-first principle. Then, the remaining unconnected contigs are linked based on the coverage weight. Finally, the combined weights between contigs—derived from summing the two weights—are output to serve as input for the connection step in Stage 4.
(4) Gap distance estimation: the gap distance between contigs is estimated based on the inter-contig weights calculated in Step 3, thereby completing contig connection and ultimately generating the genome assembly result.

### Hypergraph construction

PHScaffolding algorithm initiates by aligning Pore-C reads to contigs using the Falign [35] tool. To minimize noise from repetitive sequences and alignment errors while ensuring data reliability and computational efficiency, we rigorously filter alignments through:

(1) Alignment quality control: retaining only alignments *l_len_* > 100 with *q* ≥ 50, *l_len_* denotes the aligned length, while *q* represents mapping quality.
(2) Unique alignment removal: discarding Pore-C records aligned exclusively to single contigs, as they lack inter-contig interaction data.

After the filtering steps, we obtain a high-quality alignments to construct a weighted hypergraph *H* = (*V*, *E*). The node set *V* = {*v_1_*, *v_2_*, …, *v_n_*}, each node represents a contig. The hyperedge set *E* = {*e_1_*,*e_2_*,…,*e_m_*}. Each hyperedge *e_i_*∈*E* represents a Pore-C read, which can link multiple contigs. Hence, *e_i_* can be represented by {(*v_i1_*, *l_i1_*), (*v_i2_*, *l_i2_*), …, (*v_in_*, *l_in_*)}, where *v_ij_*∈*V* denotes the *j*-th contig, and *l_ij_* specifies the alignment length on contig *v_ij_*, *n* is the number of the contigs for *e_i_*.

By precisely retaining contig coverage lengths, this model establishes a high-confidence graph structure for hypergraph clustering. For a hyperedge *e_i_* spanning *n* nodes (linking *n* contigs), its weight *e_wj_* is calculated via the geometric mean (Equation 1) across linked contigs.


(1) \begin{equation*} e{w}_j={\left(\prod \limits_{j=1}^n{l}_{ij}\right)}^{\frac{1}{n}} \end{equation*}

### Hypergraph clustering

Following the calculation of hyperedge weights, the subsequent hypergraph clustering analysis requires two initial processing steps. First, perform an aggregation operation on the hyperedges to determine the weights between all node pairs. For instance, the weight value *w_ij_* between node contig *v_i_* and node contig *v_j_* is the sum of the weights of all hyperedges connecting both nodes. Second, apply a percentage threshold (default set to 10%) to filter *w_ij_*. This step aims to eliminate low-weight connections, thereby reducing their potential interference with the clustering results. Ultimately, this processing yields a rigorously filtered and precisely calculated weight matrix, enhancing the reliability of the subsequent clustering.

**Figure 2:**
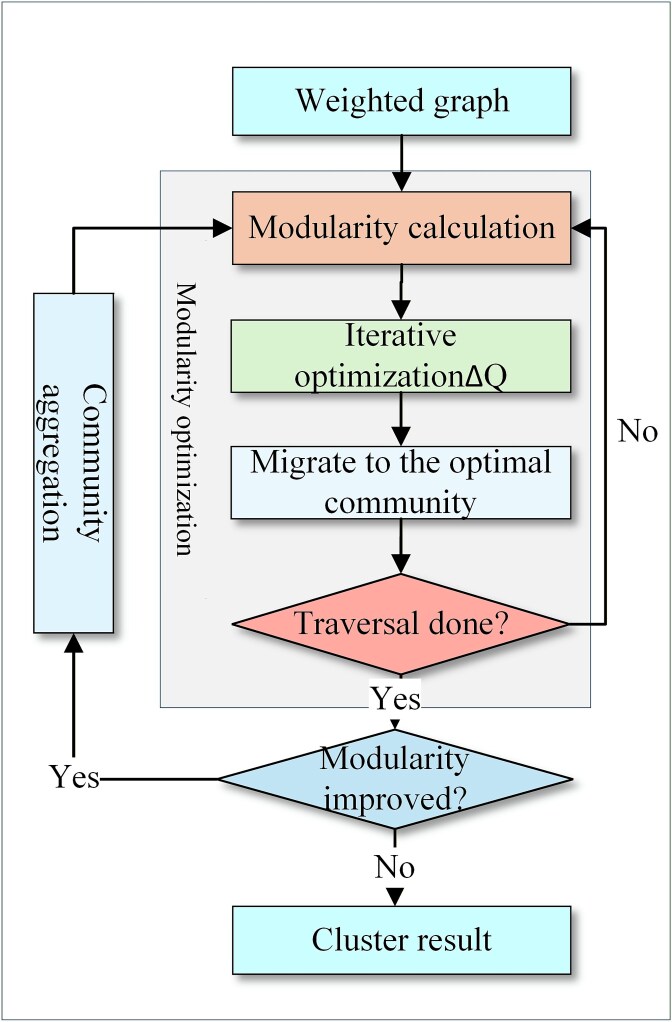
Flowchart of the Louvain algorithm.

Hypergraph clustering employs a Louvain algorithm [32], with the core objective of maximizing modularity *Q*. The algorithmic workflow is depicted in Fig. 2.

The specific steps are shown as follows:

(1) Initialization partition: each node in the weighted graph is initialized as an independent community;

(2) Modularity optimization:

a. Modularity calculation: the modularity *Q* is defined in Equation 2.


(2) \begin{equation*} Q=\frac{1}{2m}\sum \limits_{i,j}\left[{w}_{ij}-\gamma \frac{k_i{k}_j}{2m}\right]\delta \left({c}_i,{c}_j\right) \end{equation*}

where *k_i_* denotes the weighted degree of a node, equaling the sum of weights from all edges connected to it. *m* represents half the total edge weight sum in the graph. *δ*(*c_i_*,*c_j_*) denotes the community-indicator function: its value is 1 when nodes *i* and *j* belong to the same community (*c_i_* = *c_j_*), and 0 otherwise. *γ* = 1 is the resolution parameter.

b. Iterative optimization: randomly traverse all nodes. For each node *i*, calculate the modularity gain *ΔQ*(*i* → *C*) generated by moving it into each community *C*, as defined in Equation 3:


(3) \begin{equation*} \varDelta Q\left(i\to C\right)=\left[\frac{\varSigma_{\mathrm{in}}+{k}_{i,\mathrm{in}}}{2m}-\gamma{\left(\frac{\varSigma_{\mathrm{tot}}+{k}_i}{2m}\right)}^2\right]-\left[\frac{\varSigma_{\mathrm{in}}}{2m}-\gamma{\left(\frac{\varSigma_{\mathrm{tot}}}{2m}\right)}^2\right] \end{equation*}

Here, *∑_in_* represents the sum of intra-community edge weights, *∑_tot_* is the sum of weights of all edges associated with community *C* (including both intra-community edges and edges connecting *C* to other communities), and *k_i,in_* denotes the sum of connection weights between node *i* and all nodes within community *C*.

c. Node migration: for each node *i*, migrate it to the neighboring community yielding the maximum positive modularity gain *ΔQ_max_*. If all *ΔQ* ≤ 0 or *ΔQ_max_* < 10^−6^, retain *i* in its original community.

d. Convergence: repeat traversal and migration until all nodes are traversed with no migrations occurring.

(3) Community aggregation: each community optimized in the previous step is aggregated into a new community node. Based on the connection relationships between nodes in the original graph, a new weighted graph is constructed.

(4) Iteration and termination: take the newly aggregated weighted graph as input and iteratively execute steps (2) and (3) until any of the following conditions is met:

a. Modularity ceases significant improvement (*Q*^(*i* + 1)^-*Q*^(*i*)^ < 10^−6^, where *Q*^(*i*)^ is the modularity after the *i*-th aggregation);

b. Community count stabilizes;

c. Preset maximum iteration count is reached.

Upon termination, the Louvain algorithm outputs the final partition result—the community assignment for all nodes.

### Ordering and orienting

#### Determination of bins

For a contig, we only consider alignments information in its head and tail terminal regions. First, we segment five consecutive bins in its head and tail terminal regions respectively, the length of each bin is fixed.

The terminal region partitioning rules are defined as follows:

(1). If the length of a contig exceeds 10 bins, the leftmost 5 bins are designated as the head bins, and the rightmost 5 bins as the tail bins.

(2). If the contig length is greater than 5 bins but less than 10 bins, the head and tail bins are still defined as in a, allowing these regions to overlap.

(3). If the contig length is less than 5 bins, for example, if a contig is only 2.5 bins length, the head bins consists of the leftmost two full bins plus half of the adjacent bin, and the tail bins consists of the rightmost two full bins plus half of the adjacent bin.

These rules partition terminal regions of each contig to head bins and tail bins, establishing the foundation for subsequent ordering and orienting.

#### Weight calculation between contigs

After obtaining the head bins and tail bins of the contigs, we calculate weights between contigs. There are four linking types for two contigs, tail–head linking, tail–tail linking, head-tail linking, head–head linking. Tail–head linking means the tail bins of a contig can be linked by the head bins of another contig.

For two contigs, contig *a* and contig *b*, we use the previous step to obtain the head and tail bins. For each linking type, this study implemented two weighting methods to calculate its final weight: terminal coverage weight, and cosine similarity weight. Additionally, parameters for terminal coverage (*α*) and cosine similarity (*β*) can be set by users.

For the tail–head linking of contig *a* and contig *b*, if a Pore-C read can link tail bins of *a* and head bins of *b*, we get the number of bins (*C_a_*) in tail bins of *a* which can be covered by the fragments of this Pore-C read, and get the number of bins (*C_b_*) in head bins *b* using the same way.

The terminal coverage weight (*w_cov_*(*a*,*b*)) between tail bins *a* and head bins *b* for a given Pore-C read is calculated as shown in Equation 4. After computing this weight for all Pore-C reads connecting tail bins *a* and head bins *b*, these values are summed to obtain the total terminal coverage weight *W_cov_*(*a*,*b*) between the two bins.


(4) \begin{equation*} {w}_{\mathrm{cov}}\left(a,b\right)=\sqrt{C_a\times{C}_b}\times \alpha \end{equation*}

The subsequent step involves calculating the cosine similarity weight. When a Pore-C read links tail bins a and head bins b, its coverage vectors within their bins are denoted as *U_a_* and *U_b_*. The coverage vector is *U_a_* = (*u*_*a*,*0*_,*u*_*a*,*1*_,…,*u*_*a*,*5*_), where its components *u_a,i_* are defined by Equation 5. The vector *U_b_* is computed in the same manner. The cosine similarity weight *(w_sim_*(*a*,*b*)) between tail bins *a* and head bins *b* for a given Pore-C read is calculated as defined in Equation 6. After computing the cosine similarity for all Pore-C reads connecting tail bins *a* and head bins *b*, these values are summed to yield the total cosine similarity weight *W_sim_*(*a*,*b*) between the two contigs.


(5) \begin{equation*} {u}_{a,i}=\left\{\begin{array}{ll}1& \mathrm{if}\ \mathrm{the}\ {i}^{\mathrm{th}}\ \mathrm{bin}\ \mathrm{is}\ \mathrm{covered}\\{}0& \mathrm{otherwise}\end{array}\right. \end{equation*}

(6) \begin{equation*} {w}_{\mathrm{sim}}\left(a,b\right)=\frac{U_a\cdot{U}_b}{\left\Vert{U}_a\right\Vert\;\left\Vert{U}_b\right\Vert}\times \beta \end{equation*}

Following the calculation of the terminal coverage weight and the cosine similarity weight between tail bins a and head bins b, these two weights serve as the basis for the subsequent ordering and orientation of contigs.

Using the same previous steps, we can obtain the weights for all linking types between contigs.

#### Ordering and orienting contigs

First, the head and tail bins of each contig are first treated as two independent vertices in a graph. An internal edge is added between the two vertices, indicating that these two vertices belong to a the same contig. Subsequently, all cosine similarity weights between contigs are sorted in descending order, and only edges with a weight greater than or equal to 15 are retained. Following this, each remaining edge is traversed according to the sorted sequence. An edge is added if both vertices it connects have a degree less than 2 (meaning each is only connected by one internal edge) and if adding this edge does not form a cycle in the graph. This process is repeated until no new edges can be added. After connections based on cosine similarity are established, these links are preserved. Subsequently, all terminal coverage weights between contigs are sorted in descending order, retaining only edges with a weight greater than or equal to 10. The same connection strategy is then applied to supplementarily connect the contigs that were not previously linked. Through this procedure, A weighted graph is constructed, all vertices are eventually connected to form chains. Each chain is then traversed, and if an abrupt change in edge weight is encountered—specifically, if the ratio of the current edge weight to the previous one falls below a set threshold—the edge is disconnected. Following the inspection, the final ordering and orientation results are output, and the total inter-contig weight—calculated as the sum of the cosine similarity and terminal coverage weights—is provided for subsequent gap region prediction.

### Gap distance estimation

Using the obtained weight file, this study predicted and connected the gap distances between contigs based on Formula 7. Specifically, in the formula, *gap* represents the predicted inter-contig gap distance, *W_total_* denotes the total weight between contigs, *s* is the intercept parameter, and *ξ* is the decay exponent. To estimate these two parameters, the following training experiment was designed.


(7) \begin{equation*} gap={\left(\frac{10^{\log_{10}(s)}}{W_{total}}\right)}^{\frac{1}{\xi }} \end{equation*}

The study revealed [36, 37] that the total weight (*W_total_*) between most contig pairs and their gap distance follows a power-law relationship (Formula 8), where *s* is a constant and *ξ* is the decay exponent. To facilitate fitting via linear regression, base-10 logarithms were applied to both sides of the equation, transforming it into a linear form (Formula 9) Using *log₁₀*(*gap*) as the independent variable (*x*) and *log₁₀*(*W_total_*) as the dependent variable (*y*) for all contig pairs, ordinary least squares (OLS) linear regression was performed. This directly yielded estimates of the decay exponent *ξ* and the intercept *s*, which serve as the basis for predicting true inter-contig gap distances.


(8) \begin{equation*} {W}_{total}=s\times ga{p}^{-\xi } \end{equation*}

(9) \begin{equation*} {\log}_{10}\left({W}_{total}\right)=-\xi \cdot{\log}_{10}(gap)+{\log}_{10}(s) \end{equation*}

The predicted gap distance is derived by applying the inverse power-law function (Formula 7) to the estimated decay exponent *ξ* and intercept *s*, where *W_total_* represents the total inter-contig weight.

The HG002 reference genome was randomly fragmented into contigs, and the actual gap distances were recorded in a gap file. After aligning Pore-C reads to these contigs, the total inter-contig weights were calculated using the previously described ordering and orientation workflow. The weights, along with the gap file, were used as input for a pre-trained model to estimate the final parameters, *s* and *ξ*.

## Results

### Evaluation metrics

This study employs the QUAST evaluation tool to assess assembly results, utilizing the following metrics for comparison: Contig Number, NA50, NGA50, Misassemblies, Relocations, Translocations, Inversions, and Misassembled contigs. Here, Contig Number denotes the number of contigs in the assembly result, while Misassemblies refer to assembly errors including Relocations, Translocations, and Inversions. NA50 and NGA50 are critical metrics in genome assembly quality assessment, measuring assembly continuity and completeness. NA50 is derived from the alignment of assembled sequences to a reference genome. To calculate NA50, the assembled contigs are aligned to the reference, and the aligned blocks are sorted in descending order of length. The NA50 value corresponds to the length of the shortest block for which the cumulative length of all blocks of that size or larger reaches at least 50% of the total assembly length. This metric reflects the continuity of the aligned regions within the assembly. In contrast, NGA50 is calculated based on the reference genome length. The NGA50 is defined as the length of the shortest block such that the cumulative length of these blocks covers at least 50% of the reference genome length.

### Datasets

To evaluate the performance of PHScaffolding, this study employed five datasets: three HG002 datasets with 12,302 (Dataset 1), 6818 (Dataset 2), and 1296 contigs (Dataset 3), respectively; one GM12878 dataset containing 3663 contigs (Dataset 4); and one *A. thaliana* dataset comprising 5197 contigs (Dataset 5).

In this study, we utilized Pore-C reads with 21× sequencing depth for HG002, 42× for GM12878 and 60× for *A. thaliana*.

The comparison results of the five datasets against the reference are shown in Table 1.

**Table 1:** Details of datasets.

	Dataset 1	Dataset 2	Dataset 3	Dataset 4	Dataset 5
NA50	1800019	3616192	7558744	2538154	106408
NGA50	1702509	3398548	7337915	2188079	103318
Misassemblies	8061	10839	6692	5949	2676
Relocations	6476	9999	6009	3294	1575
Translocations	1520	812	669	2597	1097
Inversions	65	28	14	58	4
Misassembled contigs	4423	2004	839	1447	630
Contigs	12302	6818	1269	3653	5197

As shown in the Table 1, the Dataset 1 is characterized by shorter contig length and lower continuity, while the Dataset 2 contains more errors. These two datasets are suitable for evaluating the performance of the scaffolding method when handling fragmented contigs and elevated error rates. The Dataset 3 demonstrates the highest quality and is appropriate for assessing assembly performance on high-quality data. The Dataset 4 and Dataset 5 can be used to validate the robustness of the method across different individuals.

### Effectiveness of Louvain algorithm

#### Comparison of different weight calculation methods

This study utilized the geometric mean to calculate hyperedge weights, as this method effectively mitigates the influence of outliers. During the data alignment process, potential issues such as small duplicated regions, alignment errors, or uneven coverage—where some loci exhibit extensive aligned regions while others show minimal alignment—can distort the true confidence of hyperedges. The geometric mean is therefore more appropriate for the objectives of this study. For comparative purposes, the arithmetic mean and harmonic mean were also introduced as alternative methods to evaluate partitioning accuracy.

Partitioning accuracy is defined as the ratio of the number of contigs derived from the majority chromosome to the total number of contigs within a single partition. For the comparative experiment, the last five chromosomes of the hg002 reference genome served as the dataset. The specific procedure was as follows: these five chromosomes were randomly fragmented into contigs, and the chromosomal origin of each contig was recorded. Pore-C reads were then aligned to these contigs. Hyperedge weights were calculated separately using three methods: geometric mean, arithmetic mean, and harmonic mean. Following contig clustering based on the weights obtained from each method, the partitioning accuracy of the three approaches was statistically analyzed and compared. The results are presented in Table 2, which shows that the geometric mean method achieved the highest partitioning accuracy.

**Table 2:** Evaluation metrics of different weight calculation methods.

	Geometric mean	Arithmetic mean	Harmonic mean
Contigs	851	851	851
Number of clusters	22	19	10
Correct contigs	719	713	667
Highest accuracy	36.08%	35.32%	38.46%
Lowest accuracy	100%	100%	100%
Overall accuracy	84.49%	83.78%	78.38%

#### Clustering ablation experiment

While the ordering and orientation approach alone can accomplish genome assembly, it requires calculating weights between all contig pairs, which is computationally intensive. Furthermore, alignment errors and extensive repetitive regions can compromise assembly accuracy. In contrast, the hypergraph clustering method leverages the high-dimensional information from Pore-C sequencing reads to perform clustering, ensuring that contigs within each partition originate from the same chromosome. This approach also significantly reduces the computational time required for the subsequent ordering and orientation step. To validate the effectiveness of the hypergraph clustering method, an ablation experiment was conducted using Dataset 1, Dataset 2, and Dataset 3. The experimental results are summarized in Table 3.

**Table 3:** Experimental comparison of clustering methods.

	With clustering			Without clustering		
	Dataset 1	Dataset 2	Dataset 3	Dataset 1	Dataset 2	Dataset 3
NA50	3979701	4407075	7719802	3979701	4407075	7719802
NGA50	3669088	4079566	7568211	3679654	4079566	7568211
Misassemblies	7061	11938	6873	8067	11938	6895
Relocations	5687	11014	6155	6259	11014	6162
Translocations	1337	892	703	1766	892	718
Inversions	37	32	15	42	32	15
Misassembled contigs	3011	2013	757	3445	2013	742
Contigs	7998	5150	1055	8541	5150	1036
Times	13m08s	10m18s	9m52s	15m32s	12m14s	10m23s

### Comparison of different bin length

During the ordering and orientation phase, this study employs multi-scale bins to calculate the weight between contigs. To validate the feasibility of our approach, Dataset 2 was utilized for experimental validation, as summarized in Table 4. Here, ‘Short Bin’ refers to the assembly results obtained using only bin sizes of 400 bp, 1000 bp, and 2000 bp; ‘Long Bin’ denotes the results from using bin sizes of 5000 bp, 10 000 bp, and 20 000 bp; while ‘Short Bin + Long Bin’ represents the assembly results when all bin sizes from both categories are utilized.

**Table 4:** Evaluation metrics of different bin values on dataset 2.

	Short bin	Long bin	Short bin + long bin
NA50	3761485	4266338	4434317
NGA50	3425177	4026982	4117342
Misassemblies	10889	11561	10996
Relocations	10033	10659	11014
Translocations	826	869	896
Inversions	30	33	34
Misassembled contigs	2025	2023	2011
Contigs	6418	5593	5150

Table 4 shows that shorter bins enhance assembly accuracy but reduce contiguity, whereas longer bins improve contiguity at the cost of increased assembly errors. In this study, we integrated short and long bins by assigning a higher weight to the former and a lower weight to the latter when calculating the weight between contigs. The sum of these weights served as the final weight. Evaluation results for this combined weight are presented in the third column of Table 4. The composite weighting approach balances assembly accuracy with continuity. Thus, by assigning appropriate weights to multiple bins and summing them, this study achieved a balance between assembly accuracy and contiguity. The experimental results for Dataset 1 and Dataset 3 are provided in Supplementary Tables 1 and 2.

### Comparison of different weight strategy

Following identification of bin indices for terminal region coverage, we evaluated whether the dual-weight integration strategy could balance the accuracy and continuity of the two weighting calculations. Experimental results (Table 5) indicate that whereas cosine similarity weighting improves orientation precision, inadequate coverage points frequently induce missing weights in potential connections. In contrast, coverage count weighting—despite marginally lower precision—increases inter-node connectivity and enhances coverage. Thus, our dual-weight integration strategy ensures the accuracy of the assembly results while improving continuity. The experimental results for Dataset 1 and Dataset 3 are provided in Supplementary Tables 3 and 4.

**Table 5:** Evaluation metrics of different weight calculation methods on dataset 2.

	Cosine similarity	Terminal coverage	Integration weight
NA50	3697669	4185065	4434317
NGA50	3416309	3908780	4117342
Misassemblies	10783	11242	10996
Relocations	9939	10367	11014
Translocations	814	843	896
Inversions	30	32	34
Misassembled contigs	1979	2073	2011
Contigs	6543	5918	5150

### Comparison of connection strategies and parameter settings

Upon obtaining the cosine similarity weight and the terminal coverage weight, an initial round of connections was established using the cosine similarity weight, with all successfully connected edges retained. The terminal coverage weight was then applied to create supplementary connections among the remaining unlinked contigs. This sequential procedure was designed based on the findings from Section 3.5, which indicated that while the cosine similarity weight provides higher connection accuracy, the terminal coverage weight facilitates the linkage of a larger number of contigs. As a result, this two-stage strategy effectively balances assembly accuracy and contiguity.

Regarding the configuration of parameters α and β, this study thoroughly considered the influence of bin length on the results: although shorter bins can achieve higher accuracy, they may lead to loss of edge information; conversely, longer bins help retain more edge information but are prone to reduced assembly accuracy due to the presence of extensive repetitive regions. Accordingly, a parameter strategy was adopted in which shorter bins are assigned higher values of α and β, while longer bins are assigned lower values, thereby achieving a balance between accuracy and continuity.

To validate the effectiveness of the aforementioned connection strategy and parameter configuration, a comparative experiment was conducted on Dataset 2. Three distinct α and β configuration schemes were implemented: the differentiated configuration proposed in this study, a uniform α and β applied to all bins, and a configuration assigning higher α and β to long bins and lower values to short bins. Additionally, a fused-weight ordering and orientation scheme, which directly sums the cosine similarity weight and the terminal coverage weight, was introduced for comparison. The assembly accuracy and continuity of the different methods on Dataset 2 were compared to demonstrate the advantage of the proposed approach. The experimental results are presented in Table 6, which confirms that the strategy proposed in this study effectively balances genome assembly accuracy and continuity. The experimental results for Dataset 1 and Dataset 3 are provided in Supplementary Tables 5 and 6.

**Table 6:** Comparison of connection strategies and parameter settings on dataset 2.

	Single setting	Opposite settings	Additive strategy	Integration strategy
NA50	4236059	4013171	4149946	4434317
NGA50	3908780	3593517	3866508	4117342
Misassemblies	11721	10890	11506	10996
Relocations	10810	10045	10602	11014
Translocations	879	815	872	896
Inversions	32	30	32	34
Misassembled contigs	2065	2000	2110	2011
Contigs	5409	6350	5659	5150

### Experimental evaluation of gap distance estimation methods

To validate the effectiveness of the connection method proposed in this study, a comparative experiment was conducted on Datasets 2 and 3. The experimental design used the proposed connection method as the test group and a control scheme that sets all contig gaps to a default of 500 bp. A comparison of the results, presented in Table 7, shows that the proposed method significantly improves both the connection accuracy and continuity of the assembly.

**Table 7:** Experimental evaluation of gap distance estimation methods.

	Fixed gap length		Gap length prediction	
	Dataset 2	Dataset 3	Dataset 2	Dataset 3
NA50	4407075	7678822	4434317	7719802
NGA50	4079566	7551803	4117342	7568211
Misassemblies	11938	6887	10996	6873
Relocations	11014	6169	11014	6155
Translocations	892	703	896	703
Inversions	32	15	34	15
Misassembled contigs	2013	759	2011	757
Contigs	5150	1055	5150	1055

### Comparison with other methods

To evaluate the performance of the proposed method, a comparative analysis was conducted against three scaffolding tools—SALSA2, YaHS, and EndHiC—across all five datasets (Dataset 1 to Dataset 5). The alignment results for Dataset 1 are presented in Table 8, and those for Dataset 2 are reported in Table 9. Correspondingly, the results for Dataset 3, Dataset 4, and Dataset 5 are provided in Tables 10, 11, and 12, respectively.

**Table 8:** Evaluation metrics on dataset 1.

	SALSA2	YaHS	EndHiC	PHScaffolding
NA50	2995176	2169746	3015741	3979701
NGA50	2792826	2085933	2833058	3669088
Misassemblies	5138	9421	5298	7061
Relocations	4099	7616	4225	5687
Translocations	1019	1733	1052	1337
Inversions	20	72	21	37
Misassembled contigs	3522	3688	3484	3011
Contigs	10668	9828	12159	7998

**Table 9:** Evaluation metrics on dataset 2.

	SALSA2	YaHS	EndHiC	PHScaffolding
NA50	4145886	4460555	3616192	4434317
NGA50	3900407	4125654	3398548	4117342
Misassemblies	11133	11333	10850	10996
Relocations	10255	10484	10006	11014
Translocations	845	821	816	896
Inversions	33	28	28	34
Misassembled contigs	1886	1958	2004	2011
Contigs	6272	6125	6818	5150

**Table 10:** Evaluation metrics on dataset 3.

	SALSA2	YaHS	EndHiC	PHScaffolding
NA50	7719802	7708782	7558744	7719802
NGA50	7568502	7568211	7337915	7568211
Misassemblies	6879	6861	6684	6873
Relocations	6155	6170	5993	6155
Translocations	706	677	677	703
Inversions	18	14	14	15
Misassembled contigs	742	744	839	757
Contigs	1064	1134	1269	1055

**Table 11:** Evaluation metrics on dataset 4.

	SALSA2	YaHS	EndHiC	PHScaffolding
NA50	2708702	2739174	2567750	2646506
NGA50	2439533	2492542	2348769	2425379
Misassemblies	5126	5188	4528	5153
Relocations	4231	4353	3718	4294
Translocations	833	767	750	792
Inversions	62	68	60	67
Misassembled contigs	981	853	1329	1001
Contigs	2770	2770	3653	2660

**Table 12:** Evaluation metrics on dataset 5.

	SALSA2	YaHS	EndHiC	PHScaffolding
NA50	111996	116638	106408	119099
NGA50	107938	111045	103318	112119
Misassemblies	2828	2806	2676	3212
Relocations	1678	1690	1575	1982
Translocations	1125	1112	1097	1183
Inversions	25	4	4	47
Misassembled contigs	439	357	630	338
Contigs	4727	4857	5197	3187

NA50 and NGA50 are key metrics for assessing scaffold continuity, with higher values indicating longer sequences and better assembly quality. PHScaffolding outperformed or was comparable to other methods on these metrics across multiple datasets. In Dataset 1, both NA50 (3 979 701) and NGA50 (3 669 088) achieved by PHScaffolding were significantly higher than those of other methods, surpassing the second-best performer, EndHiC, by approximately 32% and 29%, respectively. For Dataset 2, the NA50 (4 407 075) and NGA50 (4 079 566) of PHScaffolding were comparable to those of YaHS, but higher than those of SALSA2 and EndHiC. In Dataset 3, PHScaffolding achieved the highest NA50 (7 719 802), tied with SALSA2, and the highest NGA50 (7 568 211), tied with YaHS. For Dataset 5 (*A. thaliana*), the NA50 (119 099) and NGA50 (112 119) of PHScaffolding were considerably higher than those of other methods. PHScaffolding only produced slightly lower NA50 and NGA50 values than YaHS in Dataset 4 (GM12878), but it still did better than EndHiC. Overall, PHScaffolding demonstrated consistent and superior performance in terms of continuity, particularly on human genomic data (Datasets 1–3) and *A. thaliana* data, indicating its effectiveness in generating longer and more continuous scaffolds. In terms of misassembly, the total number of Misassemblies produced by PHScaffolding was generally comparable to those of other methods across most datasets. However, the key metric of Misassembled contigs offers a more granular perspective: PHScaffolding yielded the lowest number of Misassembled contigs (3011) in Dataset 1, as well as the lowest number (338) in Dataset 5. Regarding specific misassembly types—such as Relocations, Translocations, and Inversions—PHScaffolding generally produced numbers similar to other methods. Moreover, the absolute values for certain types, such as Inversions, were small, suggesting a limited impact on overall quality. Therefore, while PHScaffolding does not exhibit a distinct advantage in misassembly reduction, its performance remains competitive. More importantly, it produces more complete and continuous assemblies, with Misassemblies being more concentrated. One of the primary objectives of scaffolding is to reduce the number of contigs by merging smaller contigs into larger scaffolds, thereby simplifying the assembly. PHScaffolding consistently produced the fewest contigs across all datasets, demonstrating its superior integration capability. In Dataset 1, PHScaffolding yielded significantly fewer contigs (7998) than other methods (e.g. 10 668 by SALSA2), representing a 35% integration rate from the initial 12,302 contigs. In Dataset 2, the contig count achieved by PHScaffolding (5150) reflected a 24% reduction from the initial 6818 contigs, outperforming the modest reductions seen with other methods. In Dataset 3, PHScaffolding produced slightly fewer contigs (1055) than SALSA2 (1064). Similarly, in Datasets 4 and 5, PHScaffolding resulted in the lowest number of contigs (2660 and 3187, respectively). This consistent reduction in contig numbers indicates that PHScaffolding identifies and joins contigs more efficiently, thereby facilitating downstream analyses. Across all evaluated datasets, PHScaffolding shows notable strengths in continuity and contig integration—key criteria for scaffolding performance. It also performs comparably to existing methods in terms of misassembly rates, while its lower count of misassembled contigs implies that errors tend to be more localized and potentially correctable in subsequent polishing. Importantly, PHScaffolding consistently achieves high continuity and assembly completeness without compromising accuracy across datasets from diverse organisms, underscoring its robustness. The method demonstrates exceptional performance in Dataset 1, indicating a particular advantage when handling complex and fragmented contig datasets. These results support the conclusion that PHScaffolding produces more contiguous and accurate assemblies across a range of genomic contexts.

Of the existing methods, SALSA2, YaHS, and EndHiC are unable to scaffold directly from Pore-C reads; they first require aligning and converting the reads into compatible formats—a process involving multiple preprocessing steps. In our comparative experiments, alignment and format conversion using the Pore-C Snakemake tool took 3–4 days for the conversion step alone. By contrast, the method proposed here employs the Falign tool to perform integrated alignment and assembly, completing the full workflow in only 4–5 hours. Thus, for alignment and assembly using the more accurate Pore-C data, PHScaffolding not only drastically reduces processing time and simplifies operation but also delivers high completeness, continuity, and accuracy in the final assembly.

## Discussion

We introduce PHScaffolding: a Pore-C-based method combining hypergraph clustering with multi-scale weighted fusion for ordering and orienting contigs. This approach efficiently extracts scaffolding signals from Pore-C alignments. Experimental results show that PHScaffolding performs robustly across all tested datasets, including those from different species, with outstanding continuity and assembly completeness alongside high accuracy.

Despite PHScaffolding’s performance across multiple datasets is well, two limitations persist. First, the method exclusively utilizes Pore-C data for scaffolding and cannot integrate genomic data such as HiFi, restricting its applicability in multi-data integration scenarios. Secondly, the connection phase, which follows ordering and orientation, estimates the sizes of inter-contig gaps using parameters obtained from training and the connection weights generated in the previous step. This method is founded solely on the power-law relationship linking gap distance and connection weight and intentionally omits more sophisticated mathematical models that could potentially yield more precise gap estimates. Moreover, owing to the constraints imposed by the limited training data, this aspect of the framework necessitates further optimization to advance the continuity and accuracy of the assembled sequences.

Future work will address these limitations. First, to enhance data adaptability, we will develop multi-source data fusion mechanisms by optimizing algorithmic frameworks. Second, to refine connection precision, gap-length prediction models will be established using genomic features and alignment information. The use of this gap-filling method significantly enhances assembly accuracy, thereby improving the overall assembly quality.

## Conclusion

This paper presents a hypergraph clustering and dual-weight integration strategy for scaffolding with Pore-C reads, aimed at addressing the challenge of assembling contigs into chromosome-level genome sequences. PHScaffolding utilizes higher-order Pore-C reads as input and employs a hypergraph to store alignment information between Pore-C reads and contigs, facilitating the clustering of contigs from the same chromosome. Prior to clustering, the hypergraph is analyzed, and a hyperedge weight calculation method is designed to determine weights between nodes. Clustering is performed using the Louvain algorithm. Subsequently, the terminal regions of contigs are partitioned using bins of different lengths, and a combined weight between nodes within each cluster is computed by integrating terminal coverage and cosine similarity. These weights are then used for ordering and orientation. The final step involves connecting the ordered and oriented sequences to produce the assembly output. Compared to existing Hi-C-based methods, PHScaffolding demonstrates enhanced accuracy and continuity, especially when handling longer contig sets. It also demonstrates broad applicability, exhibiting strong performance across diverse biological datasets. However, several challenges and future directions remain to be explored, such as the integration of additional data types for scaffolding and improvements to the gap prediction method in the final connection phase. These aspects present promising avenues for further research.

**Key Points**

- Scaffolding is essential for assembling high-quality, chromosome-level genome sequences, with significant implications for research on species diseases, genetic variation, growth mechanisms, and related biological processes.
- Based on Pore-C higher-order reads, a novel scaffolding method is proposed.
- To evaluate the performance of PHScaffolding, it was compared with the two other scaffolding methods, and a series of comparative experiments were conducted on real datasets from HG002, GM12878, and *Arabidopsis thaliana*.

## Supplementary Material

Supplementary_Materials_bbag003

## Data Availability

Dataset 1 is available from https://www.ncbi.nlm.nih.gov/datasets/genome/GCA_001542345.1/. Dataset 2 is available from https://www.ncbi.nlm.nih.gov/datasets/genome/GCA_004796285.1/. Dataset 3 is available for from https://www.ncbi.nlm.nih.gov/datasets/genome/GCA_018398555.1/. Dataset 4 is available from https://pan.baidu.com/s/1yu_SGjKuLtlO76oWZzJXog?pwd=pphs. Dataset 5 is available from https://www.ncbi.nlm.nih.gov/datasets/genome/ GCA_001742845.1/. HG002 Pore-C data is available from https://pan.baidu.com/s/1bzoKFu9uYVQZlQpsyUX--A?pwd=d8tm. GM12878 Pore-C data is available from https://www.ncbi.nlm.nih.gov/sra/?term=SRR19920180. *A. thaliana* Pore-C is available from https://download.cncb.ac.cn/gsa/CRA005105/CRR327363/.
