# Supplementary material for: PHScaffolding: a hypergraph clustering and dual-weight integration strategy for scaffolding with Pore-C reads
Source: Brief Bioinform. 2026 Jan 22;27(1):bbag003. doi: 10.1093/bib/bbag003 (PMC12825295; doi:10.1093/bib/bbag003)
Supplement: Supplementary_Materials_bbag003 [file supplementary_materials_bbag003.docx]

**Supplementary Materials**

**Implementation Details**

PHScaffolding is a scaffolding assembly tool. To use PHScaffolding, the following dependencies are required: Python 3.8.12, NumPy 1.24.4, SciPy 1.10.1, and a g++ compiler environment. All experimental datasets are hosted at https://github.com/Suquana/PHScsffolding-Dataset. The source code and detailed operating instructions for this study are available at <https://github.com/Suquana/PHScaffolding>.

**Command Lines**

PHScaffolding:

Modify the contig names in the contig file to facilitate assembly (requires modifying the file paths inside).

Python gaiming.py

Python main.py [-h] --paf PAF --contig CONTIG --output_dir OUTPUT_DIR [-q Q] [--min_w MIN_W] [--drop_w DROP_W] [-r R] [--gap GAP] -s number -e number -z weight file

Below are the parameters for running these script:

| **Option** | **Description** |
| --- | --- |
| **-h** | show this help message and exit |
| **--paf** | Input PAF file |
| **--contig** | Input contig FASTA file |
| **--output_dir** | Output directory for all result files |
| **-q** | Mapping quality |
| **--min_w** | Minimum edge weight for Ordering |
| **--drop_w** | Weight drop threshold for Ordering |
| **-r** | Resolution parameter for Louvain algorithm |
| **--gap** | Number of Ns between contigs in scaffold sequences |
| **-s** | intercept s |
| **-e** | the estimated decay exponent ξ |
| **-z** | weight file |

YaHS:

yahs [options] <contigs.fa> <hic.bed>|<hic.bam>|<hic.bin>

Below are the parameters for running these script:

| **Option** | **Description** |
| --- | --- |
| **-a** | AGP file (for rescaffolding) |
| **-r** | list of resolutions in ascending order |
| **-e** | restriction enzyme cutting sites |
| **-l** | minimum length of a contig to scaffold |
| **-q** | minimum mapping quality |
| **--read-length** | read length |
| **-o** | prefix of output files |
| **-v** | verbose level |
| **--version** | show version number |
| **-?** | print long help with extra option list |

SALSA2:

run_pipeline.py [-h] -a ASSEMBLY -l LENGTH -b BED [-o OUTPUT] [-O] [-c CUTOFF] [-g GFA] [-e ENZYME] [-i ITER] [-x DUP] [-s EXP] [-m CLEAN] [-f FILTER] [-p PRNT]

Below are the parameters for running these script:

| **Option** | **Description** |
| --- | --- |
| **-a** | Path to initial assembly |
| **-h** | show this help message and exit |
| **-e** | Restriction Enzyme used for experiment |
| **-l** | Length of contigs at start |
| **-b** | Bed file of alignments sorted by read names |
| **-c** | Minimum contig length to scaffold, default=1000 |
| **-g** | GFA file for assembly |
| **-i** | do not do memory check at runtime |
| **-x** | File containing duplicated contig information |
| **-s** | Expected Genome size of the assembled genome |
| **-o** | Output directory to put results |
| **-f** | Filter bed file for contigs present in the assembly |

EndHiC

endhic.pl hifiasm.fa.len human_100000_abs.bed human_100000.matrix human_100000_iced.matrix
Below are the parameters for running these script:

| **Option** | **Description** |
| --- | --- |
| **CONTIG_LEN_FILE** | A two-column file: contig_id and contig_length |
| **ABS_BED** | Bin BED file generated by HiC-pro |
| **RAW_MATRIX** | Raw matrix file generated by HiC-pro |
| **ICED_MATRIX** | Normalized (iced) matrix file generated by HiC-pro |
| **--binsize** | Bin size for contig end regions |
| **--binnum** | Number of bins used for each contig end |
| **--rounds** | Number of iterative rounds in endhic_iterate.pl |
| **--binnumstep** | Increment of contig-end bin number at each iteration |

## **4. Tables**

**Table 1. Evaluation Metrics of Different Bin Values on Dataset 1**

|  | Short Bin | Long Bin | Short Bin + Long Bin |
| --- | --- | --- | --- |
| NA50 | 3336901 | 3371812 | 3979701 |
| NGA50 | 2995500 | 3146344 | 3669088 |
| Misassemblies | 5553 | 7138 | 7061 |
| Relocations | 4486 | 5804 | 5687 |
| Translocations | 1043 | 1293 | 1337 |
| Inversions | 24 | 41 | 37 |
| Misassembled contigs | 3438 | 3007 | 3011 |
| Contigs | 9985 | 8425 | 7998 |

**Table 2. Evaluation Metrics of Different Bin Values on Dataset 3**

|  | Short Bin | Long Bin | Short Bin + Long Bin |
| --- | --- | --- | --- |
| NA50 | 7607209 | 7678822 | 7719802 |
| NGA50 | 7503740 | 7551803 | 7568211 |
| Misassemblies | 6722 | 6868 | 6873 |
| Relocations | 6027 | 6139 | 6155 |
| Translocations | 681 | 714 | 703 |
| Inversions | 14 | 15 | 15 |
| Misassembled contigs | 829 | 771 | 757 |
| Contigs | 1226 | 1077 | 1055 |

**Table 3. Evaluation metrics of different weight calculation methods on Dataset 1.**

|  | Cosine similarity | Terminal coverage | Integration weight |
| --- | --- | --- | --- |
| NA50 | 3084797 | 3377103 | 3979701 |
| NGA50 | 2861165 | 3191460 | 3669088 |
| Misassemblies | 5174 | 5796 | 7061 |
| Relocations | 4149 | 4651 | 5687 |
| Translocations | 996 | 1107 | 1337 |
| Inversions | 29 | 38 | 37 |
| Misassembled contigs | 3349 | 3213 | 3011 |
| Contigs | 10381 | 9446 | 7998 |

**Table 4. Evaluation metrics of different weight calculation methods on Dataset 3.**

|  | Cosine similarity | Terminal coverage | Integration weight |
| --- | --- | --- | --- |
| NA50 | 7558744 | 7607209 | 7719802 |
| NGA50 | 7337915 | 7503740 | 7568211 |
| Misassemblies | 6703 | 6714 | 6873 |
| Relocations | 6001 | 6022 | 6155 |
| Translocations | 688 | 678 | 703 |
| Inversions | 14 | 14 | 15 |
| Misassembled contigs | 838 | 835 | 757 |
| Contigs | 1261 | 1244 | 1055 |

**Table 5. Comparison of Connection Strategies and Parameter Settings on Dataset 1**

|  | Single Setting | Opposite Settings | Additive Strategy | Integration Strategy |
| --- | --- | --- | --- | --- |
| NA50 | 3793731 | 3102589 | 3908682 | 3979701 |
| NGA50 | 3476561 | 2861165 | 3603358 | 3669088 |
| Misassemblies | 5985 | 8654 | 6390 | 7061 |
| Relocations | 4783 | 7089 | 5119 | 5687 |
| Translocations | 1168 | 1526 | 1224 | 1337 |
| Inversions | 34 | 39 | 47 | 37 |
| Misassembled contigs | 2955 | 2806 | 2867 | 3011 |
| Contigs | 9257 | 7152 | 8809 | 7998 |

**Table 6. Comparison of Connection Strategies and Parameter Settings on Dataset 3**

|  | Single Setting | Opposite Settings | Additive Strategy | Integration Strategy |
| --- | --- | --- | --- | --- |
| NA50 | 7708782 | 7607209 | 7608529 | 7719802 |
| NGA50 | 7558744 | 7503740 | 7536027 | 7568211 |
| Misassemblies | 6786 | 7219 | 6742 | 6873 |
| Relocations | 6090 | 6447 | 6041 | 6155 |
| Translocations | 682 | 757 | 687 | 703 |
| Inversions | 14 | 15 | 14 | 15 |
| Misassembled contigs | 807 | 545 | 819 | 757 |
| Contigs | 1161 | 729 | 1203 | 1055 |
